# Report of Clinic on Pellagra Held in Dallas, Texas, in Connection with Meeting of Southern Medical Association, November 8-11, 1915

**Published:** 1915-12

**Authors:** John R. Lehmann

**Affiliations:** Dallas


### Report of Clinic on Pellagra Held in Dallas, Texas, in Connection With Meeting of Southern Medical Association, November 8-11, 1915.

BY JOHN R. LEHMANN, A. B., M. D., DALLAS.

Following the meeting of the Southern Medical Association in Dallas, Texas, a pellagra clinic was held on Saturday, November 13, 1915, at the Baylor University School of Medicine. This clinic was one of the series of clinics given under the auspices of the Dallas physicians and surgeons for any of the visiting members of the fraternity who desired to attend. It grew out of a movement originating in the Dallas Medical and Surgical Society. At the regular meeting in August, 1915, a committee was appointed by the President, Dr. O. M. Marchman, consisting of Dr. E. S. Fortner and Dr. H. Leslie Moore, both of Dallas, the former to investigate the etiology and the latter the prevention and treatment of pellagra. Dr. W. L. Allison, of Fort Worth, Texas, was asked to report on pellagra in the State of Texas. These reports were given as a part of the symposium on pellagra held in connection with the sessions of the medical section of the Southern Medical Association, which met November 8-11, 1915.

The symposium, with its live discussions, and the clinic which followed vindicate the organization and perpetuation of the Southern Medical Association. Its avowed aim is to stimulate the interest of Southern physicians to a study and solution of the medical problems peculiar to their section of the United States. It is fair to say that nowhere ever in the South has there been a more enthusiastic and comprehensive scientific discussion of the pellagra problem than there was at this time. Intellectual steel and flint were working every minute during these sessions. The world at large will soon feel the heat of the flame of investigation kindled there.

It is estimated that there are in Texas at present from 40,000 to 45,000 cases of pellagra. And the estimated death rate annually from this dread disease is 500. These estimates were made after correspondence with many physicians throughout the State. This correspondence was carried on by a few interested members of the profession, and the above statistics compiled from replies to their letters.

In connection with the study of pellagra in the United States, mention should be made of the splendid work of those Texas physicians who have authoritatively acquainted the medical world in Europe as well as America with the nature, prevalence and ravages of the malady. High among these stands Dr. K. H. Beall, of Fort Worth, the author of the chapter on pellagra in Osler's System of Medicine.

A report of the clinic mentioned above is hereto appended. There were in attendance about ninety medical men and twenty patients, showing different phases of pellagra, were presented for study. A number of short discussions with the cases as illustrative were a feature of the clinic.

Dr. Homer Bruce, Opelika, Ala., spoke on the "Etiology of Pellagra." Pellagra among the better classes, he holds, occurs in those invalided and who, therefore, do not eat enough of the proper form of food to keep them up in vital resistance. He does not agree with Dr. Goldberger in his theory as to etiology, nor has he seen enough cases of associated amebiasis to lead him to pin much faith to this theory.

The gastro-intestinal phases of pellagra were discussed by Dr. H. G. Walcott, of Dallas. He has seen about one hundred cases of pellagra with gastro-intestinal symptoms prominent. In these, when gastric analysis was made, which was not done in every instance, there was almost always achylia gastrica. There is a characteristic odor to the stool. Two cases showed amebiasis. Trichomonads do not occur exclusively in the diarrhea of pellagra.

On the skin lesions in pellagra, Dr. J. B. Shelmire, of Dallas, reported his observations. These depend upon the severity and duration of the dermatitis. Scaling begins in eight or ten days after onset of eruption. Sometimes the latter begins with an edematous condition. The glove of pellagra does not extend as high on the flexor aspect as on the extensor. The first manifestation may be only on the points of the elbows—pigmentation and roughness. The collar is less common in this country than in Europe. One case seen by him showed the dermatitis about the genitalia alone. In the majority of cases the eruption begins as macules, which spread, simulating sunburn and progressing to ulceration.

The hands of those not working manually look like those of the laboring classes. They are roughened and discolored. The palms are exempt. Don't jump at the conclusion that a case is pellagra from the skin symptoms alone.

Dr. R. W. Baird, of Dallas, brought out some points on the etiology of pellagra. "I used to think the etiological factor was a one-sided diet. Now I am inclined to consider the disease an infection. Diet, however, does have something to do with the malady. Those, in well-to-do families, who develop pellagra, are they who eat whimsically, largely of carbohydrates, to the exclusion of proteins. I had a patient who went to Colorado. She was getting on well with a general diet when she entered the Battle Creek institution at Boulder, Colo., where you know they are vegetarians. She soon developed acute pellagra and died."

A case, with discussion of the treatment of the condition, was presented by Dr. C. M. Grigsby, of Dallas.

"This patient complains of burning of the hands and feet. The dermatitis was worse in spring. He has had mental disturbance—poor memory. Also had diarrhea. He has improved, but still has stomatitis. He has had sodium cacodylate, in the way of treatment.

"Salts and thymol in treatment do not seem to do much good. I doubt whether any disease, wasting, such as pellagra and pernicious amenia, which shows a tendency to spontaneous improvement, can be cured at all. I shall treat my cases in the future along Goldberger's suggestions. There is no definitely known cure."

Dr. Gengenbach, of Denver, Colo., made some remarks on pellagra in children in Denver. He told about a boy, 20 years old, who had been living in the South. He had come to Colorado. He had pellagra, and was then in extremis. Dr. Gengenbach was called to see him. He soon died. Children have not had the one-sided diet so long as adults. Hence, if this has an effect in its production, it has not had as long a time to act. There is no pellagra indigenous to Colorado. The cases seen there are those imported from the Southern States, coming or being sent to the cooler climate of this State.

"The Lower Intestinal Symptoms of Pellagra" was the subtopic handled by Dr. B. Kinsell, of Dallas. He reported one case seen by him with diarrhea, pain in the rectum and stomach symptoms. These disappeared under treatment. Rectal symptoms were the first in his cases. He has seen three other cases with prominent proctitis, foul discharge and mucous erythema.

"Some Aspects of Pellagra" was taken up by Dr. W. L. Allison, of Fort Worth, Texas. "Pellagra is a good deal more prevalent than we think. It is a disease of the central nervous system. The eruption is always symmetrical, unless the nerve supply be impaired peripherally or centrally, then it appears only on the side whose nerve supply is intact. Generally the eruption is a late manifestation. I have seen pellagra bloom out following an operation. The typical pellagra mouth is never seen in any other disease except pellagra. Intense burning somewhere in the body is a dependable symptom, may be in the stomach, on the feet, hands or face. History of stomach trouble for years, with loss of weight, insomnia, parathesias—spells pellagra. Let us be constantly on the lookout for the disease. I do not have the least doubt that many pellagrins recover permanently. I believe it is a toxemia. Many toxemias recover without any treatment. Diet has nothing to do with pellagra, except to affect vitality. In some ways, pellagra is much like malaria. There is no specific treatment. We must be urgent in our therapeutic insistence that the patients stay with the treatment for a long period of time. Rest is very valuable—it is absolutely essential to put them to bed. I like sodium cacodylate, grains 5, once a day for six days, then skipping a few days and beginning over again. Hydrochloric acid is valuable. I give dilute hydrochloric, ten to fifteen minims, three times a day, one-half hour after meals. As a mouth wash, I use potassium chlorate and citrate solution. Large doses of bismuth, one teaspoonful every second hour, will control in many cases a diarrhea that hydrochloric will not handle."

Dr. Allison thinks pellagra is an insect-borne, protozoan disease.

DISCUSSION.

Dr. Hayes, of Mississippi: "I had pellagra in my family, a little daughter, who was cared for by a negro mammy, who, two years later, died of pellagra. She was a cornbread fiend; ate it herself and fed the child on it. Most cases I have seen in the better classes declare that they do not eat meat, can't stand eggs, eat mostly carbohydrates, such as cereals," etc.

Dr. J. A. Hammack, Kennedale, Texas: "Dr. Goldberger is very close to the mark. There is something which we take into our mouths that cause it—as cornbread."

Dr. M. M. Smith, Dallas, Texas, thinks that penury, with its very protein-poor and variety-lacking diet, is a potent factor.

Dr. Dunn, Burleson, Texas, has seen but two cases die of pellagra. "I cannot get interested in the cornbread theory. In Jackson county they don't eat cornbread as they used to forty years ago. Everybody has cows: it is a dairy and beef section, and yet they have pellagra. We have used cacodylate of sodium." —*Texas Medical News.*

